# Handgrip strength as a potential indicator of aging: insights from its association with aging-related laboratory parameters

**DOI:** 10.3389/fmed.2025.1491584

**Published:** 2025-01-29

**Authors:** Nina Kemala Sari, Stepvia Stepvia, Muhana Fawwazy Ilyas, Siti Setiati, Kuntjoro Harimurti, Ika Fitriana

**Affiliations:** ^1^Geriatric Division, Department of Internal Medicine, Faculty of Medicine, Dr. Cipto Mangunkusumo National Referral Hospital, Universitas Indonesia, Jakarta, Indonesia; ^2^Faculty of Medicine, Universitas Sebelas Maret, Surakarta, Indonesia; ^3^Clinical Epidemiology and Evidence-Based Medicine Unit, Dr. Cipto Mangunkusumo National Referral Hospital, Jakarta, Indonesia

**Keywords:** aging, biomarkers, elderly population, functional capacity, handgrip strength

## Abstract

**Introduction:**

The aging process is frequently associated with a decline in functional capacity, endurance, muscle quality, and overall quality of life. Examining aging-related biomarkers often requires significant time and financial resources, underscoring the need for a straightforward and practical indicator. This study aims to investigate the association between handgrip strength and aging-related laboratory parameters in the elderly population of Indonesia.

**Methods:**

A cross-sectional study was conducted involving 109 participants aged 60–82 years. Handgrip strength was measured using a Jamar hydraulic hand dynamometer and Jamar PLUS+ digital dynamometer. Aging-related laboratory biomarkers were defined as those indicating physiological aging processes.

**Results:**

The study revealed a significant association between handgrip strength and several aging-related laboratory parameters, including leukocyte count, absolute neutrophil count, absolute lymphocyte count, neutrophil/lymphocyte ratio, and erythrocyte sedimentation rate.

**Discussion:**

These findings suggest that handgrip strength could serve as a cost-effective, non-invasive predictor of aging-related health status in older adults. Its practical utility highlights its potential for guiding health interventions targeting the elderly population.

## 1 Introduction

Globally, the aging population is rapidly increasing, posing significant challenges for healthcare systems. According to the World Health Organization (WHO), the number of people aged 60 years or older will rise from 900 million to 2 billion between 2015 and 2050 (moving from 12 to 22% of the total global population) (1). This demographic shift is accompanied by a rise in age-related health issues, such as cardiovascular disease, metabolic disorders, and degenerative illnesses (2). Consequently, there is a growing need for efficient and effective markers to assess and manage older adults’ health and functional capacity. In Indonesia, an increasing challenge with its aging population also appeared, projected to rise from 13% in 2019 to nearly 20% by 2045 (3). Data from the Central Statistical Authority of Indonesia show a 5–10% increase in health problems from 2020 to 2022. Therefore, it is crucial to continue advanced research on the capacity and identification of frailty-related ailments, for instance, by focusing on portable strength as an indicator of aging (4–8).

The aging process is often associated with a reduced ability to perform and endure physical activities, including a decline in muscle quality (7). Evaluating essential motor characteristics, such as muscle strength, is necessary when assessing dependence and physical performance in older individuals (8). Research indicates that the decline in muscle function associated with aging can serve as an initial indication of various concurrent or long-term health concerns, such as cardiovascular disease, metabolic disorders, cerebrovascular illness, and susceptibility to falls resulting in injuries (9–12). A study in Jakarta revealed that only 2% of advanced-stage cancer patients had normal muscle strength, highlighting the potential direct correlation between muscle strength and comorbidities (13). Subsequently, another study has shown a clear link between age-related health issues and handgrip strength assessments using tools like the SphygmoCor and Jamar Dynamometer (14).

Reduced muscular strength, as assessed using handgrip strength assessments, has also been associated with an increased risk of mortality due to cardiovascular disease. Handgrip strength is considered a simple, quick, and cost-efficient technique for classifying an individual’s likelihood of experiencing cardiac death (15, 16). The decreased handgrip strength among elderly adults is a substantial risk factor for disability, sickness, and mortality. Additionally, it also plays a vital role in the diagnosis of sarcopenia and frailty (17). Handgrip strength measurements can also be beneficial in identifying patients who are at a heightened risk of frailty and require more treatment and care (18). Subsequently, it has also proved to be a powerful diagnostic tool for detecting vulnerabilities in aged individuals with hematological (19) and degenerative disorders (20).

In recent years, aging-related biomarkers have gained attention for their potential to provide insights into the physiological changes that accompany aging. Biomarkers such as erythrocyte sedimentation rates (ESRs), lymphocyte counts, erythrocytes, platelets, and neutrophil-lymphocyte ratios (NLRs) have been proposed as indirect indicators of aging due to their links to inflammation, immune function, and overall systemic health. These biomarkers offer a means to monitor changes in body function and structure that may correlate with age-related health decline (21–23).

ESR reflects the acute phase protein concentration and serves as a marker of inflammatory activity. However, this test is not specific to any particular disease and often increases with age due to chronic low-grade inflammation, a phenomenon sometimes referred to as “inflammaging.” This chronic inflammation is linked to increased susceptibility to degenerative diseases and frailty in older adults (24–26). Similarly, lymphocyte levels and NLR provide insights into immune response and resilience, with studies suggesting that immune cell ratios may reflect the body’s ability to respond to stressors and infections as it ages. As people age, their immune system undergoes significant changes, particularly in the number and types of lymphocytes present in the bloodstream. In older individuals, there is a decrease in the number of CD4+ T cells, CD8+ T cells, and B lymphocytes. Conversely, the number of natural killer (NK) lymphocytes tends to increase. These changes can make older adults more susceptible to infectious diseases and increase the likelihood of negative health outcomes when lymphocyte counts are low (27–29).

Aging also leads to a progressive dysregulation of immune functions, affecting all types of leukocytes, including T cells, B cells, monocytes, and macrophages (30). Leukocyte Telomere Length (LTL) has been recognized as a biomarker for aging. Shorter telomeres in leukocytes correlate with increased biological age and are associated with cognitive decline and other age-related health issues (31). Neutrophils, the most abundant type of white blood cells, play a crucial role in the innate immune response and have increasingly been considered potential biomarkers of aging (32). Aged neutrophils exhibit distinct phenotypic markers; they show decreased expression of L-selectin (CD62L) and increased expression of CXCR4. Additionally, neutrophils from older individuals tend to be hyper-responsive, contributing to chronic low-grade inflammation—a hallmark of “inflammaging” (33).

Studies by Nevill et al. (34) and his team have discovered that the number of erythrocytes, lymphocytes, and thrombocytes are positively associated with handgrip strength (14, 34, 35). This study revealed that optimal numbers of lymphocytes, erythrocytes, and platelets promote muscular tissue growth and may increase handgrip strength. Subsequently, several factors, including age, gender, ESR, NLR, body mass, and height, have also been interrelated and can influence physical performance and muscular strength (14, 20, 34, 35). Therefore, a simple predictor that can precisely explain other predictors of aging is needed. Including these hematological biomarkers alongside measures of physical function, for instance, handgrip strength allows for a multidimensional assessment of aging-related health status, incorporating both musculoskeletal and systemic health indicators. Therefore, this study aims to investigate the relationship between handgrip strength and aging-related laboratory parameters in the elderly population of Indonesia.

## 2 Methods

### 2.1 Study design

This cross-sectional study was conducted on community-dwelling older adults from the Cempaka Putih Community Health Center and Johar Baru Community Health Center, Jakarta, Indonesia, between July and August 2023. To ensure thorough and transparent reporting, this study adheres to the STROBE (Strengthening the Reporting of Observational Studies in Epidemiology) checklist guidelines for observational research. A purposive sampling approach was used to select participants based on eligibility criteria. Participants aged 60 or older were eligible if they had no diagnosed upper limb pathology, pain in the hand, wrist, or forearm, or prior neurological conditions affecting the upper quadrant. Exclusion criteria included lack of informed consent, sensory impairments, finger or hand amputations, active arthritis, hemiplegia, quadriplegia, recent upper extremity surgery (within the last 3 months), or acute infection or symptoms. The study analyzed socio-demographic data, pre-existing medical disorders, physical measurements, and laboratory biomarkers. Subsequently, handgrip dynamometers were used to assess muscle strength by measuring handgrip strength. The Ethics Committee of the Faculty of Medicine, University of Indonesia–Dr. Cipto Mangunkusumo National Referral Hospital gave ethical approval to carry out the study (Ethical Application Ref: 23-11-1936 and date of approval: 15 March 2023).

### 2.2 Measurement of the handgrip strength

The JAMAR^®^ hydraulic hand dynamometer [Model J00105, Lafayette Instrument Company, United States of America (United States)] and the JAMAR^®^ PLUS+ digital dynamometer [Model 0814006453, Sammons Preston Company, United States of America (United States)] were used to evaluate the handgrip strength of the dominant hand following the previous study procedure (36). This device relies on a hydraulic mechanism rather than load cells or springs, allowing it to accurately measure grip force through fluid compression. Prior to the study, the dynamometer was calibrated using certified weights to ensure measurement accuracy and reliability. The researchers tested the study participants’ handgrip strength while sitting down, with their elbows placed next to their body and bent at a 90-degree angle. Their wrist was positioned in a neutral alignment, and a supportive surface was placed beneath the dynamometer. The participant must apply maximum force to compress the handgrip strength dynamometer during exercise. Handgrip strength quantification entails measuring the static force the hand applies when compressing or squeezing a dynamometer. The maximum voluntary contraction was sustained for 3 s and quantified as the handgrip strength in kilograms (kg). Three readings were taken at 60-s intervals, and the highest and average reading was chosen for analysis.

### 2.3 Measurement of the aging-related laboratory parameter

Blood samples for laboratory tests were collected at the time of participant recruitment to ensure consistency across all participants. To minimize variability, samples were obtained under standardized conditions following an 8-h fasting period, ensuring recent food intake or other factors did not influence the results. The aging-related laboratory parameters, analyzed as biomarkers of aging, were measured using well-established methods in recognized laboratories. Aging-related laboratory parameter tests included a thorough analysis of relevant blood components, including hemoglobin, hematocrit, leukocyte count, erythrocyte count, platelet count, basophil count, eosinophil count, neutrophil count, lymphocyte count, and monocyte count. All samples underwent complete blood counts using a Hematology Analyzer (Alinity H, Abbott Laboratories, United States). Additional calculations included the NLR calculated from the analyzer’s output and ESR measured using the Westergren method.

### 2.4 Statistical analysis

All statistical analysis was performed using IBM SPSS Statistics software version 25 (IBM Corp., Armonk, NY, United States). First, univariate analysis was performed. The continuous variables are expressed as mean and standard deviation (SD) values and their normality distribution is assessed using the Kolmogorov–Smirnov test. The categorical variables are presented using frequency and percentages. Subsequently, the differences within gender groups were examined utilizing the Independent *T*-test or Mann–Whitney for continuous variables and Chi-square or Fisher’s exact test for categorical variables. Last, a partial correlation analysis, adjusted for gender and age, was performed to investigate the relationship between handgrip strength and aging-related laboratory parameters. This analysis included bootstrapping with 100 samples, using simple random sampling and a 95% confidence interval. *p*-values less than 0.050 were considered statistically significant.

## 3 Results

### 3.1 Participant characteristics

This study included a cohort of 109 elderly populations in Indonesia aged between 60 and 82 years living in the community. The mean age, height, and weight were 66.2 ± 5.3 years, 152.0 ± 7.4 centimeters, and 58.1 ± 9.9 kilograms, respectively. The mean BMI was 25.1 ± 3.7 Kg/m^2^. The major comorbidities observed in the subjects are as follows: hypertension (28.4%), rheumatology disease (19.3%), diabetes mellitus (16.5%), and hypercholesterolemia (9.2%). Subsequently, the differences in study participants’ characteristics are stated in Table 1. In characteristics, there is a significant difference in height (*p* < 0.001) and BMI (*p* = 0.024) between different genders; however, no significant differences were found in other parameters.

**Table 1:** Characteristics of study participants.

Characteristics	Total (*n* = 109)	Female (*n* = 85)	Male (*n* = 24)	*p*-value
Age (years)	66.25 ± 0.506	65.86 ± 0.554	67.63 ± 1.178	0.162^b^
Height (cm)	151.96 ± 0.706	149.515 ± 0.592	160.633 ± 1.380	<0.001*^b^
Weight (kg)	58.06 ± 0.952	57.22 ± 1.061	61.06 ± 2.062	0.095^a^
BMI (kg/m^2^)	25.09 ± 0.359	25.52 ± 0.415	23.57 ± 0.620	0.024*^a^
Hypertension (n)	31 (28.4%)	24 (28.2%)	7 (29.2%)	0.929^c^
Rheumatology disease (n)	21 (19.3%)	16 (18.8%)	5 (20.8%)	0.777^d^
Diabetes mellitus (n)	18 (16.5%)	13 (15.3%)	5 (20.8%)	0.540^d^
Hypercholesterolemia (n)	10 (9.2%)	8 (9.4%)	2 (8.3%)	1.000^d^
Gastrointestinal disease (n)	8 (7.3%)	8 (9.4%)	0 (0.0%)	0.196^d^
Hyperuricemia (n)	6 (5.5%)	6 (7.1%)	0 (0.0%)	0.335^d^
Sarcopenia (n)	3 (2.8%)	3 (3.5%)	0 (0.0%)	1.000^d^
Cerebrovascular disease (n)	3 (2.7%)	3 (3.5%)	0 (0.0%)	1.000^d^
COPD (n)	2 (1.8%)	1 (1.2%)	1 (4.2%)	0.393^d^
Liver disease (n)	2 (1.8%)	2 (2.4%)	0 (0.0%)	1.000^d^
Vertigo (n)	2 (1.8%)	1 (1.2%)	1 (4.2%)	0.393^d^
Hernia nucleus pulposus (n)	2 (1.8%)	2 (2.4%)	0 (0.0%)	1.000^d^
Coronary heart disease (n)	1 (0.9%)	1 (1.2%)	0 (0.0%)	1.000^d^
Glaucoma (n)	1 (0.9%)	1 (1.2%)	0 (0.0%)	1.000^d^
Cancer (n)	1 (0.9%)	1 (1.2%)	0 (0.0%)	1.000^d^

Data are presented as mean ± SD for continuous variables or frequency (precentage) for categorical variables. ^a^Independent *T*-test; ^b^Mann-Whitney; ^c^Chi-square or ^d^Fisher’s exact test were performed. *p-value < 0.05 indicates statistical significance.

Tables 2, 3 display the aging-related laboratory parameters as well as the mean and maximum value of handgrip strength among study participants. The analysis revealed significant gender differences in several aging-related laboratory parameters and handgrip strength measures. Males had notably higher hemoglobin (*p* < 0.001), hematocrit (*p* = 0.010), eosinophil absolute counts (*p* = 0.037), and monocyte absolute counts (*p* = 0.022) compared to females. Additionally, males exhibited lower platelet counts (*p* = 0.045). Handgrip strength was also significantly higher in males across all measures, including both manual and digital assessments, as well as both mean and maximal values (*p*-values of < 0.001).

**Table 2:** Aging-related laboratory parameters of study participants.

Measurement	Total (*n* = 109)	Female (*n* = 85)	Male (*n* = 24)	*p*-value
Hemoglobin (g/dL)	13.327 ± 0.118	13.123 ± 0.126	14.050 ± 0.246	<0.001*^b^
Hematocrit (%)	38.561 ± 0.344	38.052 ± 0.360	40.367 ± 0.811	0.010*^b^
Leukocyte (10^3^/μL)	7.580 ± 1.182	7.565 ± 0.206	7.633 ± 0.394	0.655^b^
Erythrocyte (10^6^/μL)	4.453 ± 0.050	4.401 ± 0.052	4.635 ± 0.134	0.101^b^
Platelets (10^3^/μL)	288.633 ± 9.244	296.635 ± 10.755	260.292 ± 16.766	0.045*^b^
Basophil absolute (10^3^/μL)	45.505 ± 2.221	44.118 ± 2.404	50.417 ± 5.399	0.312^b^
Eosinophil absolute (10^3^/μL)	200.000 ± 12.608	187.412 ± 13.345	244.583 ± 31.236	0.037*^b^
Neutrophil absolute (10^3^/μL)	4611.284 ± 144.490	4635.765 ± 165.281	4524.583 ± 302.430	0.971^b^
Lymphocyte absolute (10^3^/μL)	2188.349 ± 64.043	2191.176 ± 71.244	2178.333 ± 147.685	0.895^b^
Monocyte absolute (10^3^/μL)	568.991 ± 47.552	500.000 ± 14.426	813.333 ± 205.430	0.022*^b^
Neutrophil/Lymphocyte ratio	2.266 ± 0.092	2.260 ± 0.102	2.287 ± 0.218	0.971^b^
Erythrocyte sedimentation rate (mm/h)	51.890 ± 2.076	53.682 ± 2.220	45.542 ± 5.095	0.105^a^

Data are presented as mean ± SD. ^a^Independent *T*-test or ^b^Mann-Whitney were performed. *p-value < 0.05 indicates statistical significance.

**Table 3:** Hand grip strength of study participants.

Measurement		Total (*n* = 109)	Female (*n* = 85)	Male (*n* = 24)	*p*-value
Manual	Mean value (kg)	19.678 ± 0.610	17.714 ± 0.477	26.633 ± 1.518	<0.001*^a^
	Maximal value (kg)	21.083 ± 0.642	19.000 ± 0.496	28.458 ± 1.604	<0.001*^b^
Digital	Mean value (kg)	24.630 ± 0.631	23.142 ± 0.564	29.900 ± 1.685	<0.001*^b^
	Maximal value (kg)	27.027 ± 0.677	25.259 ± 0.588	33.287 ± 1.764	<0.001*^b^

Data are presented as mean ± SD. ^a^Independent *T*-test or ^b^Mann-Whitney were performed. *p-value < 0.05 indicates statistical significance.

### 3.2 Association between handgrip strength and aging-related laboratory parameters

Partial correlation analysis, adjusted for gender and age, revealed significant associations between handgrip strength and various aging-related laboratory parameters (Table 4). Leukocyte counts were inversely correlated with handgrip strength in manual assessments (*r* = −0.237 to −0.239, *p* < 0.05). The neutrophil count also displayed a strong inverse correlation across both manual (*r* = −0.405, *p* < 0.001) and digital (*r* = −0.256 to −0.261, *p* < 0.05) assessments. In contrast, lymphocyte count showed a positive correlation with handgrip strength across all measurements (*r* = 0.268–0.286, *p* < 0.01). Additionally, the NLR was negatively associated with handgrip strength, reaching significance in both manuals (*r* = −0.494 to −0.501, *p* < 0.001) and digital assessments (*r* = −0.371 to −0.388, *p* < 0.001). Last, ESR was weakly but significantly correlated with digital mean handgrip strength (*r* = 0.224, *p* = 0.020) and maximum strength (*r* = 0.201, *p* = 0.038).

**Table 4:** Correlation between hand grip strength and aging-related laboratory parameters.

Variables	Hand grip strength							
Aging-related laboratory parameters	Manual				Digital			
	Mean		Maximum		Mean		Maximum	
	*r*	*p*	*r*	*p*	*r*	*p*	*r*	*p*
Hemoglobin (g/dL)	−0.006	0.952	0.002	0.985	−0.019	0.847	−0.010	0.921
Hematocrit (%)	−0.047	0.633	−0.037	0.702	−0.048	0.625	−0.035	0.720
Leukocyte (10^3^/μL)	−0.239	0.013*	−0.237	0.014*	−0.110	0.260	−0.105	0.284
Erythrocyte (10^6^/μL)	−0.056	0.569	−0.052	0.592	−0.051	0.599	−0.061	0.529
Platelets (10^3^/μL)	−0.177	0.069	−0.158	0.104	0.028	0.772	0.017	0.864
Basophil absolute (10^3^/μL)	−0.147	0.130	−0.155	0.110	0.015	0.881	0.023	0.817
Eosinophil absolute (10^3^/μL)	0.024	0.805	0.056	0.564	0.055	0.575	0.070	0.476
Neutrophil absolute (10^3^/μL)	−0.405	<0.001*	−0.405	<0.001*	−0.256	0.008*	−0.261	0.007*
Lymphocyte absolute (10^3^/μL)	0.272	0.005*	0.271	0.005*	0.268	0.005*	0.286	0.003*
Monocyte absolute (10^3^/μL)	0.028	0.772	0.018	0.853	0.102	0.296	0.087	0.374
Neutrophil/Lymphocyte ratio	0.501	<0.001*	−0.494	<0.001*	−0.371	<0.001*	−0.388	<0.001*
Erythrocyte sedimentation rate (mm/h)	0.096	0.325	0.100	0.304	0.224	0.020*	0.201	0.038*

The correlation between variables was analyzed using the partial correlation test, which was adjusted by gender and age. *p-value < 0.05 indicates statistical significance.

## 4 Discussion

Currently, aging-related laboratory parameters are increasingly suggested as biomarkers for assessing aging in older adults. These biomarkers serve as prognostic instruments, evaluating disease progression and the efficacy of interventions (37). This study observed significant associations between handgrip strength and several hematological parameters, specifically leukocyte, neutrophil absolute, lymphocyte absolute, NLR, and ESR.

Handgrip strength measures the force exerted by the fingers and thumb, reflecting the individual’s capacity for strength, endurance, and functional ability (38). In addition to its use in musculoskeletal assessment, handgrip strength provides a versatile indicator relevant to dynamic physical tasks and, as a clinical measure, is often assessed using bedside dynamometers such as the Jamar Hydraulic Hand Dynamometer (39–41). Subsequently, it has shown correlations with a range of medical conditions, including chronic anemia, dyslipidemia, metabolic syndrome, chronic renal disease, hypertension, and cardiovascular diseases (40, 42). Additionally, a longitudinal study has demonstrated that low handgrip strength correlates strongly with risks of type 2 diabetes (43). Decreased handgrip strength frequently indicates reduced muscle quality, which can lead to metabolic dysregulation and insulin resistance as people age. This could entail a decrease in skeletal muscle mass, poor functioning of mitochondria, and changes in the release of adipokines. These factors all contribute to metabolic disruptions and a deterioration in insulin sensitivity (44).

According to a meta-analysis conducted by Wu et al. (45), a reduction of 5 kg in grip strength was found to be correlated with a higher likelihood of developing cardiovascular disease. In a separate study conducted by Wei et al. (46), it was also discovered that the possibility of death from any cause and death from cardiovascular disease increased steadily as handgrip strength quartiles decreased for both men and women (47). Aging blood arteries may exhibit enhanced rigidity, reduced responsiveness to vasodilators and vasoconstrictors, and diminished angiogenesis. Subsequently, a study conducted by Kaur et al. (48) demonstrated that for each 5-kg reduction in handgrip strength, there was a 16% increase in the hazard ratio for all-cause death. Handgrip strength was also discovered to be a more powerful indicator of overall mortality and cardiovascular mortality than systolic blood pressure (48).

Handgrip strength is crucial for evaluating muscle function and is also strongly associated with sarcopenia and frailty. A study by Forrest et al. (49) discovered a notable decrease in grip strength among elderly individuals in the United States who experienced physical restrictions such as difficulty standing from a chair, walking, ascending steps, and going outdoors (49). In addition, muscle strength is correlated with bone mineral density. A comprehensive review conducted by Denk et al. (50) revealed that all 11 papers included in the analysis validated a correlation between reduced handgrip strength and the occurrence of hip fractures resulting from the combination of osteoporosis and sarcopenia, sometimes referred to as ‘osteosarcopenia’. Subsequently, age-related sarcopenia also increases the likelihood of experiencing functional impairments. Last, individuals with lower grip strength have limitations in performing instrumental activities of daily living (IADL) and activities of daily living (ADL) when compared to those with higher handgrip strength (51).

During the aging process, there is a decline in functional qualities, resulting in a loss of homeostasis and a reduced ability to adjust to internal and external stressors. This makes individuals more susceptible to disease and death (52). Aging-related muscular atrophy is a prevalent kind of muscle wasting in humans, characterized by a notable decline in muscle function, including reduced movement speed and muscle weakness. The process of muscle atrophy that occurs due to aging will result in sarcopenia, characterized by a decrease in muscle mass, strength, and function. This will eventually lead to frailty, including self-reported exhaustion, slowed performance and walking speed, weakness, unintentional weight loss, and low physical activity. These symptoms are the combined effects of various organ systems (53). Generally, to diagnose sarcopenia, it is necessary to test both muscle mass and strength and assess physical performance. Multiple imaging modalities have been utilized to evaluate muscle mass and quality, including dual-energy X-ray absorptiometry (DXA), computed tomography (CT), magnetic resonance imaging (MRI), and ultrasound (US). Regrettably, many patients may find imaging procedures to be financially inaccessible. Therefore, handgrip strength measurement could serve as a valuable method for diagnosing sarcopenia and frailty in elderly individuals (54).

Hematological markers, including leukocytes, are essential components of the immune system, and their levels and functions undergo significant changes as individuals age. Immunosenescence, a decline in immune function, happens along the process of aging. This decline affects all types of leukocytes, including T cells, B cells, monocytes, and neutrophils. For instance, studies have shown that the number of naive T cells decreases while the proportion of memory T cells increases with age, reflecting a shift in immune response capabilities (30, 55). Additionally, the expression of specific surface markers can indicate lymphocyte senescence. For example, the loss of CD28 expression is one of the most consistent markers of senescence in T cells. Aged individuals often show increased frequencies of CD28- T cells, which are associated with reduced proliferative capacity and diminished immune responses (56). NLR also undergoes significant changes in elderly populations. Specifically, the reference intervals for NLR increased significantly with age. A study showed that higher NLR levels were associated with increased odds of being frail, indicating that systemic inflammation may play a role in the development of frailty syndrome (57). Elevated levels of neutrophils relative to lymphocytes are also an indication of systemic inflammation, which is implicated in various age-related diseases such as cardiovascular disease and diabetes (58).

ESR is also a common laboratory marker that can be used to overview the process of aging among older adults. It measures how quickly red blood cells settle at the bottom of a test tube over a specified period, typically 1 h. Research indicates that ESR tends to increase with age. A study involving elderly subjects found the upper limit of ESR for males and females aged 50 years or less was suggested as 15 mm/h and 25 mm/h, respectively, and for males and females aged above 50 years was suggested as 20 mm/h and 30 mm/h (59). In older adults, a persistently high ESR (e.g., above 50 mm/h) is frequently linked to significant underlying health issues, including rheumatological diseases, cardiovascular diseases, infections, and even cancer. It is also not only a marker of acute inflammation but also serves as an indicator of chronic inflammatory states associated with aging (60).

The correlation between handgrip strength and hematological parameters, including leukocyte levels, especially neutrophil and lymphocyte counts, NLR, and ESR, may underscore handgrip strength’s value in identifying systemic health risks. An increased level of handgrip strength may influence the leukocyte value. An absolute number of neutrophils predicted diminished grip strength among the oldest and most functionally disabled, suggesting an interactive effect of age and underlying illness on the association between neutrophilia and muscle weakness (61). Subsequently, a recent systematic review and meta-analysis have found that greater handgrip strength, as well as knee extension strength, is associated with lower levels of pro-inflammatory cytokines, including interleukin-6 (IL-6) and tumor necrosis factor-α (TNF-α). These cytokines may boost neutrophil activity, potentially mediated by cortisol and the hypothalamic–pituitary axis (62). Handgrip strength is inversely associated with leptin levels in youth, affecting both independent and global measures. This association may also stimulate leukocytes and promote the release of pro-inflammatory cytokines like TNF-α and IL-6 (63).

NLR also has a strong inverse relationship with handgrip strength. NLR is a commonly used peripheral biomarker that indicates the intensity of the inflammatory response, driven by neutrophils, along with the diminishing immune-regulatory function of lymphocytes. A previous study showed that higher NLR levels suggest a greater recruitment of pro-neuroinflammatory mediators, and this effect is further intensified with advancing age (64). This is supported by a recent study that highlighted in adults aged 50 years and older, increasing NLR was associated with lower grip strength, indicating that the effects of aging on muscle function are amplified by systemic inflammation (65).

This study also indicates that higher ESR levels are associated with reduced handgrip strength in older adults. This relationship suggests that systemic inflammation may play a role in muscle weakness and functional decline within this population (26). Inflammation is thought to be an essential risk factor for sarcopenia, as it induces a catabolic state in the muscles. An ESR is also commonly linked to chronic inflammation, which can adversely affect muscle strength (66). Another possible explanation is nutritional deficiencies, which can also influence both ESR and handgrip strength. Malnutrition is prevalent among older adults and can lead to increased inflammation, reflected by elevated ESR levels, as well as decreased muscle strength indicated by low handgrip strength (67, 68).

There are several hematological markers, such as hemoglobin, platelets, and monocytes, that are found to have a significant correlation with handgrip strength in other studies. However, in our study, those hematological markers were not significantly correlated with handgrip strength. A study found that hemoglobin levels often decline in older adults, which may contribute to decreased oxygen-carrying capacity and lower physical endurance, impacting quality of life (69). While hemoglobin itself does not contribute directly to muscle healing, its supportive function in oxygen transport is crucial, especially in aging individuals who often face endurance limitations. Additionally, the occurrence of anemia in elderly individuals may further decrease muscle mass due to disuse atrophy resulting from a decline in exercise tolerance (70).

Similarly, platelet levels and function are affected by age-related inflammatory processes and cardiovascular risks (71). Oxidative stress, a known factor in aging, plays a significant role in the physiological changes observed in blood components, particularly platelets. Platelets are essential in maintaining hemostasis under normal conditions. Yet, they are susceptible to abnormal activation in the presence of inflammation, endothelial dysfunction, and oxidative stress—factors commonly associated with aging. This can lead to increased platelet aggregation, elevating the risk of thrombosis and atherosclerosis (72). In another study, monocyte counts, particularly when elevated, are also indicative of low-grade chronic inflammation—a condition commonly seen with aging and associated with conditions like sarcopenia and frailty (73). Subsequently, a recent systematic review and meta-analysis performed by Tuttle et al. (62) indicated that higher systemic inflammation is linked to lower muscle strength and muscle mass; hence, the usage of monocyte counts combined with handgrip strength could potentially be used as the marker for frailty among older adults (62).

## 5 Limitations

Despite the valuable insights provided by this study, several limitations must be acknowledged. This study’s cross-sectional nature may restrict the ability to establish causality between handgrip strength and aging-related laboratory parameters. Longitudinal studies are necessary to explore causative relationships and the directionality of associations over time. Subsequently, this study did not account for physical activity levels or nutritional intake, both of which may influence muscle strength and overall health in older adults. Lastly, this study only includes biomarkers from routine blood counts. Future research could explore additional biomarkers, such as oxidative stress and other inflammatory markers, to further enhance the predictive power of handgrip strength.

## 6 Conclusion

This study revealed a significant association between handgrip strength and aging-related laboratory parameters, including leukocyte, neutrophil absolute, lymphocyte absolute, NLR, and ESR. These findings suggest that handgrip strength can be a simple and effective predictor of aging-related health status in older adults. The ease of measuring handgrip strength with a hand dynamometer makes it a practical tool in clinical settings, potentially aiding in the early identification of individuals at risk for various age-related health conditions. However, future studies should consider additional parameters, such as inflammatory indicators and surrogate markers, to better understand the complex interactions between muscle strength and various biomarkers of aging. This broader approach would enhance the reliability and applicability of handgrip strength as a diagnostic tool in geriatric health assessments, aiding in the early detection and management of age-related health issues.

## Data Availability

The raw data supporting the conclusions of this article will be made available by the authors, without undue reservation.
